# Augmented cellular uptake and homologous targeting of exosome-based drug loaded IOL for posterior capsular opacification prevention and biosafety improvement

**DOI:** 10.1016/j.bioactmat.2022.02.019

**Published:** 2022-02-26

**Authors:** Siqing Zhu, Huiying Huang, Dong Liu, Shimin Wen, Liangliang Shen, Quankui Lin

**Affiliations:** Department of Biomaterials, School of Ophthalmology & Optometry, Eye Hospital, Wenzhou Medical University, Wenzhou, 325027, PR China

**Keywords:** Exosome, Intraocular lens, Surface modification, Targeted therapy, Posterior capsular opacification

## Abstract

Posterior capsular opacification (PCO), the most common complication after cataract surgery, is caused by the proliferation, migration and differentiation of residual lens epithelial cells (LECs) on the surface of the intraocular lens (IOL). Although drug-loaded IOLs have been successfully developed, the PCO prevention efficacy is still limited due to the lack of targeting and low bioavailability. In this investigation, an exosome-functionalized drug-loaded IOL was successfully developed for effective PCO prevention utilizing the homologous targeting and high biocompatibility of exosome. The exosomes derived from LECs were collected to load the anti-proliferative drug doxorubicin (Dox) through electroporation and then immobilized on the aminated IOLs surface through electrostatic interaction. In vitro experiments showed that significantly improved cellular uptake of Dox@Exos by LECs was achieved due to the targeting ability of exosome, compared with free Dox, thus resulting in superior anti-proliferation effect. In vivo animal investigations indicated that Dox@Exos-IOLs effectively inhibited the development of PCO and showed excellent intraocular biocompatibility. We believe that this work will provide a targeting strategy for PCO prevention through exosome-functionalized IOL.

## Highlights

• The lens epithelial cell exosomes were separated and the Dox was subsequently loaded, rendering enhanced lens epithelial cell uptaken due to the homologous targeting feature.
• The Dox loaded exosome-functionalized intraocular lens greatly improves the uptake of Dox by LECs in vitro and exhibited excellent posterior capsular opacification prevention preventive effect in vivo.
• The drug loaded lens epithelial cell exosome immobilization provides a simple and safe surface modification method for intraocular lens postoperative complications prevention.

## Graphical abstract

The lens epithelial cell exosomes were separated and used to loading antiproliferative drug for IOL surface. Due to the homologous targeting feature, such drug loaded exosome functionalized IOL shows improved drug uptaken and enhanced and targeted lens epithelial cell apoptosis inducing. The intraocular implantation shows that such exosome functionalized IOL show great PCO prevention effect with reduced reducing undesired side effects to surrounding tissues.

**Figure:**
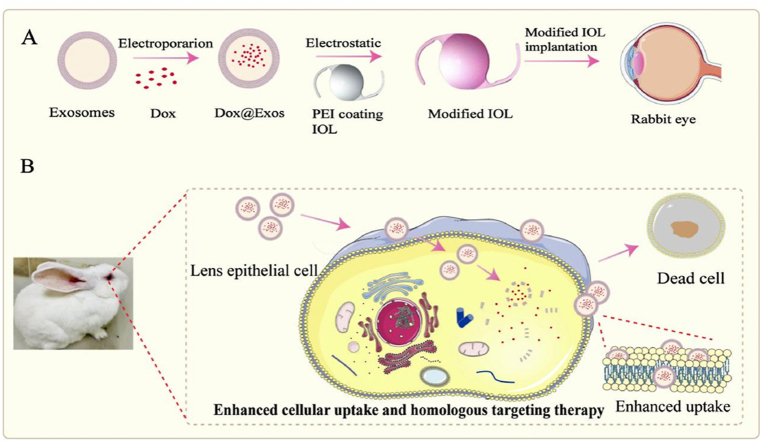

## 1 Introduction

Cataract, the opacity of lens, is still the leading cause of vision lost around the world. As the global population ages, the incidence of cataract is increasing [[1], [2], [3]]. Phacoemulsification combined with intraocular lens (IOL) implantation is the only effective treatment currently [4]. However, surgical wound healing and foreign body reactions often lead to multiple complications, among which posterior capsular opacification (PCO) is the most common complication of cataract surgery. It is reported that the incidence of PCO is 20–40% in adults and up to 100% in children within 2–5 years after surgery [5,6]. The main cause of PCO is the proliferation, migration and differentiation of residual lens epithelial cells (LECs) adhering to the IOL surface and posterior capsule [7,8]. At present, neodymium-doped yttrium aluminum garnet (Nd: YAG) laser capsulotomy is the most commonly used treatment for PCO, which, however, will cause a series of new complications, such as IOL injury and displacement, macular cystic edema and retinal detachment [9,10]. Therefore, it is of great significance to implement more efficient and safer PCO preventive measures.

At present, in addition to the optimization of IOL shape and development of novel IOL materials, IOL surface modification, which is easy to prepare and does not require extra intraocular surgery also contributes to PCO prevention [6,[11], [12], [13], [14], [15], [16], [17], [18]]. In previous studies, researchers mainly focused on hydrophilic coating on the IOL surface to prevent the adhesion of LECs [[19], [20], [21], [22]]. However, recent studies have shown that hydrophilic coating can only initially reduce LECs adhesion and proliferation on the surface but do not inhibit PCO in the long term [23]. As a result, drug loaded coating modified IOL implantations as potential treatments for posterior cataract have attracted wide attention [17,[24], [25], [26], [27]]. For example, in our previous studies, drug loaded IOLs were prepared by layer-by-layer assembly or surface-initiated reversible addition-breaking chain transfer (SI-RAFT) polymerization to prevent PCO [19,28]. In vivo experiments showed that the coating can effectively inhibit the proliferation of LECs. However, due to lack of targeting and poor bioavailability, the released drugs may potentially toxic to the surrounding intraocular tissues. Therefore, targeted drug delivery systems are urgently needed.

**Scheme 1:**
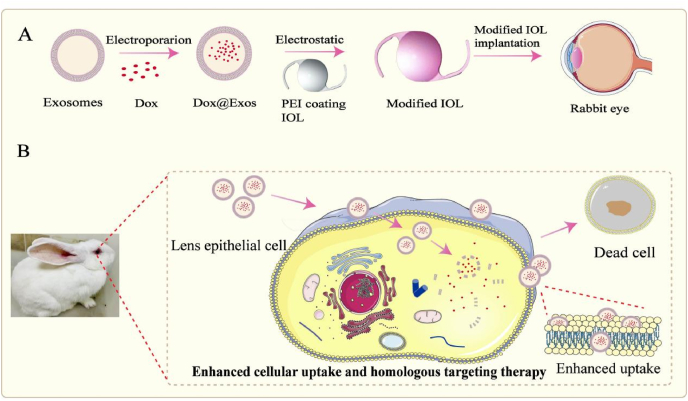
Fabrication of Dox@Exos modified IOLs and in vivo cellular uptake enhanced by homologous targeting for effective PCO prevention.

Exosomes are nanoscale extracellular vesicles secreted by cells with a diameter of 40–150 nm, and can be isolated from various body fluids and cell propagation media [[29], [30], [31]]. It is well-acknowledged that the similarity of phospholipid bilayer structure with their parental cells and specific membrane proteins and lipids on their surface can promote their fusion with parental cells, achieving targeted cellular uptake [[32], [33], [34], [35], [36], [37]]. In recent decades, exosomes as natural nanocarriers have been widely used in the diagnosis and treatment of various diseases due to high biocompatibility, low toxicity and homologous targeting [[38], [39], [40], [41], [42], [43]]. For example, Jun Wang et al. reported that exosomes derived from neutrophil can target the inflammatory site of tumor tissue more efficiently by taking advantage of neutrophil's inherent inflammatory chemotaxis [44]. As a result, it is hypothesized that targeting uptaking of anti-proliferative drug can be achieved by introducing LEC derived exosomes onto drug loaded coating on IOL surface, resulting in effective and safer PCO prevention. Herein, the exosome-based drug loaded coating on IOL surface for PCO prevention is investigated. As shown in Scheme 1, doxorubicin (Dox) loaded exosome (Dox@Exos) was firstly prepared by embedding Dox into exosomes derived from LECs by electroporation. The negatively charged Dox@Exos was then immobilized on the positively charged polyethyleneimine (PEI) pretreated IOL surface by electrostatic interaction, obtaining Dox@Exos-IOLs. It is anticipated that the exosomes can significantly improve the drug bioavailability due to their homologous targeting to parental LECs, thus enhancing the PCO prevention effect and reducing undesired side effects to surrounding tissues.

## 2 Materials and methods

### 2.1 Materials

Doxorubicin hydrochloride (Dox·HCl) was purchased from Meilunbio (Dalian, China). Polyethyleneimine (PEI, average Mn = 25,000) and DMEM/F-12 containing l-glutamine were purchased from Sigma. Fetal bovine serum (FBS), DMEM/F12 (1:1) cell culture media, 0.05% trypsin-EDTA, penicillin-streptomycin solution, and other cell culture-related reagents were purchased from Gibco (Carlsbad, USA). Hoechst 33,342, Dio, cell counting kit-8 (CCK-8), enhanced BCA protein assay (BCA) kit, and RIPA lysis buffer were purchased from Beyotime Biotechnology Co. (Shanghai, China). Phosphate-buffered saline (PBS) was purchased from Boster Biological Technology (Pleasanton, CA, USA). Polyethylene terephthalate (PET) was used as the experimental substrate in material characterization and in vitro experiments. A serial of antibodies used for western blotting (WB) were purchased from Santa Cruz. Foldable hydrophobic acrylic IOLs were supplied by 66 Vision Technology Co., Ltd. (Suzhou, China).

### 2.2 Cell culture and animals

Human lens epithelial cells (HLECs) and retinal pigment epithelium cells (RPEs) were propagated in DMEM/F-12. Human corneal epithelial cells (HCECs) were propagated in DMEM/F-12 containing l-glutamine. The culture medium was supplemented with 10% FBS and 1% penicillin-streptomycin and the cells were incubated in 5% CO_2_ at 37 °C.

Two-month-old Japanese white rabbits were obtained from Experimental Animal Center of Wenzhou Medical University. The animal experiments were carried out in accordance with the approved experimental protocol and the regulations of the Laboratory Animal Ethics Committee of Wenzhou Medical University.

### 2.3 Isolation and purification of exosomes

In order to collect exosomes, a large number of LECs in good condition were implanted in T75 culture flasks and passed for several times. When the cells reached 80–90% confluence, the original culture medium was discarded. Cells were washed 5 times with 5 mL of preheated PBS, and then 6 mL of preheated exosome solution were added into the flasks for further culture for 24 h. The medium was collected and differential centrifugation method was employed to isolate exosomes. The medium was initially centrifuged at 500 g for 10 min to remove cellular debris. Then, supernatant was carefully transferred into new tubes and then the apoptotic corpuscles were removed by centrifugation at 3000*g* for 20 min. Subsequently, millipore membrane filter (SLGP033RB, Millipore) with 0.22 μm pore size was used to remove large extracellular vesicles. Finally, 2 h of ultracentrifugation using Optiseal tubes (Beckman Coulter) and a SW32Ti ultracentrifuge rotor (Beckman Coulter) was carried out at 140,000 g to remove residual media components. All centrifugation steps were carried out at 4 °C. After discarding of the supernatant, the exosomes were resuspended into 1 mL of PBS and placed at −80 °C for long-term storage [45].

### 2.4 Labeling of exosomes

The fluorescent dye, 3, 30-dioctadecyloxacarbocyanine perchlorate (Dio), was used to label the exosome membranes. Exosomes or drug-loaded exosomes were incubated with Dio (0.05 mg/mL) for 30 min at 37 °C, then mixed with Exo-Quick Precipitation (SBI precipitant, USA) (5:1). After the mixture was incubated at 4 °C overnight in dark, and further twice centrifuged at 3000 g for 30 min to remove free dye, the labeled exosomes were resuspended in equal volume of PBS prior to use [34].

### 2.5 Fabrication and characterization of Dox@Exos

To construct Dox@Exos, 200 μL of purified exosomes were mixed with certain amount of Dox, and then electroporated in a 0.4 cm electroporation cuvette implemented at 250 V and 350 μF for 4.5 MS using a Bio-Rad Gene electroporation apparatus [34,40]. During this period, the exosome membrane was punctured to form holes for sufficient loading of Dox into exosomes, and then incubated at 37 °C for 30 min to allow recovery of the exosome membrane, followed by the addition of SBI precipitant (5:1) and standing for the night at 4 °C. In order to remove unloaded Dox, the medium was washed by cold PBS and centrifuged at 3000 g for 30 min. Above washing and centrifugal operations were carried out twice, and the purified Dox@Exos were resuspended in equal volume of PBS prior to use.

The size distribution and zeta potential of exosomes and Dox@Exos was analyzed by dynamic light scattering (DLS, Malvern Instrument Ltd, Malvern, UK). For estimating the concentration of exosomes, a BCA protein assay kit was performed according to the manufacturer [41,44]. Scanning electron microscope (SEM, Thermo Scientific, Netherlands) was used to observe the surface morphology and three-dimensional structure of exosomes and Dox@Exos. To confirm the encapsulation efficiency (EE), the absorption intensity of free Dox, exosomes and Dox@Exos at 500 nm were analyzed by a UV-spectrophotometer (UV-1780, Suzhou, China), and quantified according to a pre-determined Dox standard curve. And the fluorescence spectra of different samples were also carried out to confirm the successful loading of the Dox. Afterwards, the co-localization of Dox and fluorescence-labeled exosomes in HLECs was observed by fluorescence microscopy and analyzed using Image J software (National Institutes of Health, USA).

The exosomal markers including CD9 and Mac were confirmed by Western blot analysis. Briefly, characteristic proteins of exosomes were extracted with RIPA lysis buffer, and quantified with a BCA protein assay kit. CD9 and Mac proteins on the polyvinylidene difluoride (PVDF) membranes were separated via the sodium dodecyl sulfate polyacrylamide gel electrophoresis (SDS-PAGE), blocked with PBS-Tween 20 (PBST) fat-free dried milk at 37 °C for 1 h, and then incubated with their primary anti-bodies at 4 °C overnight [46]. Subsequently, the membranes were washed by PBST five times and incubated with the secondary antibody at 4 °C for 30 min followed by another time of PBST washing. Immunoreacted proteins were visualized using a gel imaging system (Azure C300, Azure Biosystems Inc). Otherwise, the purified Dox@Exos were re-suspended in PBS and stored at 4 °C. At selected time intervals, the Dox@Exos were blown and re-suspended. Then the particle size and Zeta potential of the sample were determined by DLS over 7 days to investigate the stability of Dox@Exos.

### 2.6 Preparation of Dox@Exos-modified IOLs

As a negative charged nanoparticle, the exosomes can be immobilized onto the material surface by electrostatic interaction [34,40,47]. Herein, the Dox@Exos were immobilized onto the IOL surface by electrostatic self-assembling. The specific process for fabricating the Dox@Exos coating on IOL materials was displayed in Scheme 1. Firstly, the materials were sonicated successively in ethanol and deionized water (dH_2_O). The materials were soaked overnight in 3 mg/mL PEI aqueous solution, washed with PBS and dried with nitrogen at room temperature, generating a positively charged aminated surface. Then, the PEI-coated materials were immersed into the Dox@Exos suspension, and incubated at 4 °C for 24 h. Purified Dox@Exos-modified materials were obtained after the un-immobilized Dox@Exos were washed away with PBS.

### 2.7 Characterization of Dox@Exos-modified IOLs

The amount of the Dox@Exos immobilized on the material surfaces was determined by using a BCA protein assay kit according to the difference of protein content of exosomes in suspension before and after the surface immobilization. Surface element changes of the modified IOL materials were detected by XPS (Kalpha, Thermal VG, America). Water contact angle analysis (OCA20; Data Physics Instrument GmbH, Germany) was performed to evaluate the surface wettability. Meanwhile, the immobilized exosomes were stained with Dio to investigate their distribution on the surface of the modified materials. Stereoscopic microscopy (SMZ1500, Nikon, Japan) was used to observe the macroscopic appearance of IOLs before and after modification. SEM was used to observe the microscopic morphology. Furthermore, the optical properties of the materials were studied by UV–vis spectroscopy and refractive index. In order to further evaluate the drug release behavior of the Dox@Exos-modified IOL, the materials were incubated in 200 μL PBS (PH = 7.4), and store in a 37 °C shaker. At certain time intervals, 100 μL supernatant was taken for analysis and replaced with fresh buffer. Further, the shedding of exosomes from the Dox@Exos coating was also investigated by BCA protein quantitation. Briefly, the release buffer of the Dox@Exos coating samples at 0, 6, 12, 24, 48, 72 h were collected and the protein content was measured by standard BCA method. In addition, DLS and TEM were used to detect and observe the particle size distribution and morphology in the released liquid after cumulative release for three days.

### 2.8 Optimization of drug concentration and co-incubation time in vitro

Several factors that affect the interaction between Dox@Exos and cells were investigated, including concentration of Dox loaded in exosomes, co-incubation time and the cellular uptake efficiency. To confirm the optimal concentration of loaded Dox, HLECs were seeded into 24-well plates containing 1 mL DMEM/F-12 at a density of 3.2 × 10^4^ per well and cultured for 24 h. When the cells reached 90% confluence, the primary culture medium was removed and fresh medium composed of 1 mL DMEM/F-12 and 200 μL of Dox@Exos with different concentrations were introduced to wells, resulting in a series of final concentration (3.9 μg/mL, 7.8 μg/mL, 11.7 μg/mL, 15.6 μg/mL, 19.5 μg/mL, respectively). At the same time, free Dox equivalent to the Dox@Exos was also added into the wells as the control group. After co-incubation for another 24 h, washing with PBS and nucleus staining with Hoechst 33,342, the viability of the HLECs in the plants was assessed by cell density. CCK-8 was also conducted to further compare the anti-proliferative effect between Dox@Exos and free Dox.

In order to achieve maximum uptake and utilization of exosomes, we explored the appropriate incubation time between exosomes and HLECs. Dox@Exos was co-incubated with HLECs for 2 h, 4 h, 6 h, 8 h and 10 h, respectively. Then, fluorescence microscopy was performed to capture the fluorescence images of various groups at each time point which were then analyzed with Image J, so as to determine the optimal co-incubation time of HLECs and Dox@Exos.

### 2.9 Enhanced cellular uptake and targeting of Dox@Exos in vitro

Firstly, the HLECs were cultured in 96-well plates (1 × 10^4^ cells per well) for 24 h to ensure that the cells are in a good adherent growth state. Afterwards, the membrane-stained Dox@Exos with determined concentration was incubated with HLECs for 5 min, 30 min, 1 h, and 4 h, respectively. Free Dox equivalent to the Dox@Exos was also added into the wells as the control group. At each time point, fresh medium containing 10% Hoechst 33,342 was incubated with the HLECs in dark for 15 min. After the removal of the residual dye, the fluorescence signal profiles representing cellular uptake status were obtained on a fluorescence microscopy.

To further understand the targeting of exosomes in vitro, HLECs, RPEs and HCECs were cultivated and passaged according to a standardized cell culture protocol. The HLECs and RPEs were pre-seeded into 96-well plants containing 200 μL of DMEM/F-12 while the HCECs were propagated in DMEM/F-12 containing l-glutamine for 24 h to ensure that the cells were in a good adherent growth state. Following the steps described previously, membrane staining of Dox@Exos was performed. Dox@Exos suspension or free Dox of the same concentration were subsequently mixed with plates containing 100 μL fresh culture medium and incubated with cells for 5 min, 15 min, 30 min respectively. Then, the cells were washed with PBS and the nuclei were stained with Hoechst 33,342 for 15 min. After the excessive dye was washed away with PBS, fluorescence microscopy was used to observe the intracellular distributions of Dox and exosomes.

### 2.10 In vitro antiproliferative analysis

The anti-proliferation activity of the Dox@Exos-modified materials was evaluated by the cellular morphology and viability. The materials were placed into a 96-well cell culture plate for continuous sterilization by ultraviolet irradiation. Meanwhile, the pristine materials and Exos-modified materials as the control groups were treated in the same way. When the HLECs reached 90% confluence, the cells were collected, counted using a cell counter, distributed into the 96-well plate at a density of 5000 per well, and incubated at 37 °C under a 5% CO_2_ atmosphere for 24 h, 48 h, 72 h, respectively. After the wells were washed with PBS gently three times, the residual HLECs were stained by Hoechst 33,342 and visualized by a fluorescence microscope (Nikon Corporation, Tokyo, Japan). The density of the adherent cells of different groups was analyzed by Image J.

The cytotoxicity of exosomes and Dox@exos was revealed by a conventional Cell Counting Kit 8 (CCK-8) assay. The PBS-washed wells were fixed with fresh culture medium containing 10% CCK-8 reagent, and co-incubation with the residual cells for another 4 h. Finally, the cellular viability was calculated based on the absorbance at 450 nm, which was measured on a microplate reader.

### 2.11 Intraocular anti-PCO efficacy

To evaluate the in vivo therapeutic efficacy of the Dox@Exos-modified IOLs, PCO models were constructed on two-month-old Japanese white rabbits by phacoemulsification combined with IOL implantation. The IOLs implantation and postoperative observation were by the methods of our previous publications [15,48,49]. The rabbits were treated, monitored, and evaluated according to the Association for Visual and Ophthalmic Research guidelines. The animal experiments were approved by the Laboratory Animal Ethics Committee of Wenzhou Medical University. Before surgery, the weight and intraocular pressure of rabbits was routinely examined and slit lamp was used to exclude rabbits with congenital eye diseases and mental disorders. The right eyes used for surgery were treated with levofloxacin eye drops three times a day for three days before implantation. 9 rabbits were divided into three groups and implanted with the Dox@Exos modified IOLs (as the experimental group), exosome-modified IOLs and pristine IOLs (as the control groups), respectively. All surgeries were performed by Dr Han. During the postoperative weeks, the surgical eyes were treated with levofloxacin eye drops, tobramycin dexamethasone ointment, atropine eye drops and pranoprofen eye drops to prevent postoperative infection.

Without sedation or anesthesia, the pupils of the operative eyes were dilated and examined by slit lamp for acute postoperative ocular inflammation including corneal edema, anterior chamber exudation and inflammation after 1, 3, and 7 d. The development of PCO was observed by slit lamp at each time point. In order to investigate the in vivo biocompatibility, the morphology and number of corneal endothelial cells were investigated using a specular microscopy. The fundus morphologies were observed by fundus camera. The retina electrophysiology function was recorded by an electroretinograph (ERG). The body weight and intraocular pressure of rabbits were measured during postoperatively periods. Subsequently, the rabbits were humanely sacrificed after 27 days. The bilateral eyeballs were removed and the lens capsule, cornea, iris and retina were carefully isolated, and the isolated tissues were stained with hematoxylin-eosin staining (HE). By observing the morphology of eye tissue and the thickness of posterior capsular hyperplasia, the degree of posterior cataract can be further assessed.

## 3 Results and discussion

### 3.1 Fabrication and characterization of Dox@Exos

**Fig. 1:**
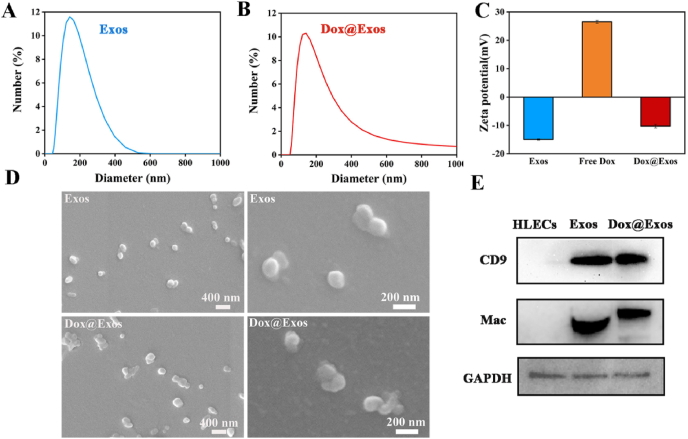
Fabrication and characterization of Exos. A-B) Distribution of hydrodynamic diameter, C) zeta potential, D) SEM images of exosomes and Dox@Exos, respectively; E) WB results of specific biomarker expression of exosomes (CD9 and Mac) in each group containing HLECs, exosomes and Dox@Exos.

Exosomes were isolated from HLECs medium in accordance with a previously reported sequential ultracentrifugation method [44]. DLS results in Fig. 1A and C indicated that the exosomes were negatively charged with a zeta potential of −13 mV and exhibited a narrow size distribution centered at 152 nm, which providing a practical basis for the mobilization of exosomes to IOL surfaces by electrostatic interactions. SEM images in Fig. 1D showed that the exosomes are uniformly dispersed accompanied with partial adhesion due to the structural characteristics of membrane. Moreover, WB results in Fig. 1E indicated the expression of exosome-characteristic proteins, such as CD9 and Mac. In general, the above results strongly indicated the successful preparation of exosomes. As previously reported, the hydrophilic lumen of exosomes can be well loaded with water-soluble drugs [35]. Then, an anti-proliferative drug Dox was loaded into exosomes by electroporation to form Dox@Exos. DLS results showed that there was a slight increase in the hydrodynamic diameter of Dox@Exos, with a peak value of 160.4 nm (Fig. 1B) whereas the surface charge maintained negatively charged (−10 mV), comparing with exosomes. Combinatorial analysis of SEM images (Fig. 1D) and Western blot results (Fig. 1E) showed that Dox@Exos still preserved the exosomal nanoscale vesicle structure and surface function proteins. In addition, when suspended in PBS buffer at 4 °C, the size of Dox@Exos showed no obvious change within 7 days, demonstrating good storage stability (Fig. S1).

**Fig. 2:**
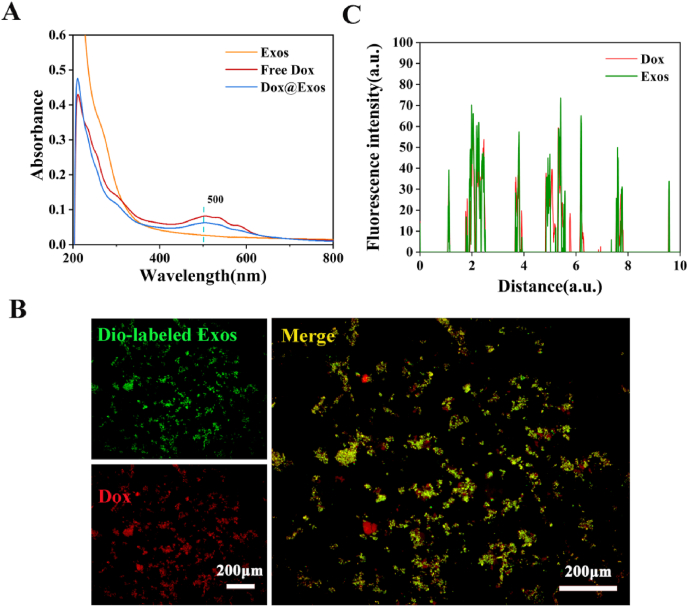
A) UV–vis spectra of exosomes, free Dox, and Dox@Exos dispersed in PBS; B) Fluorescence microscopy images of HELCs co-incubated with Dox@Exos and co-localization analysis between Dox (red) and Dio-labeled Exos (green). C) Fluorescence co-location analysis of Dio-labeled Exos and Dox.

To further confirm the successful construct of Dox@Exos, Uv absorption of free Dox, exosomes and Dox@Exos samples was measured. As shown in Fig. 2A, characteristic peaks of Dox were arising in the spectra of Dox@Exos compared with exosomes. According to the absorbance of Dox at 500 nm, quantitative analysis indicated that the Dox loading capacity was around 72%. Meanwhile, the fluorescence spectra also confirmed that Dox was successfully loaded into exosomes. Dox@Exos and free Dox showed a strong Dox fluorescence peak at 596 nm (Fig. S2). In addition, the fluorescence images in Fig. 2B exhibited the overlapping of red fluorescence of Dox and green fluorescence of Dio-labeled Exos, indicating that electroporation can greatly realize the drug loading capacity of exosomes. What's more, the corresponding semi-quantitative analysis obtained by the line scan showed a coincidence of fluorescence of Dio-labeled Exos with that of Dox (Fig. 2C), further confirming that the anti-proliferative drug was successfully encapsulated in exosomes which were in good condition.

### 3.2 Optimization of drug concentration and co-incubation time in vitro

**Fig. 3:**
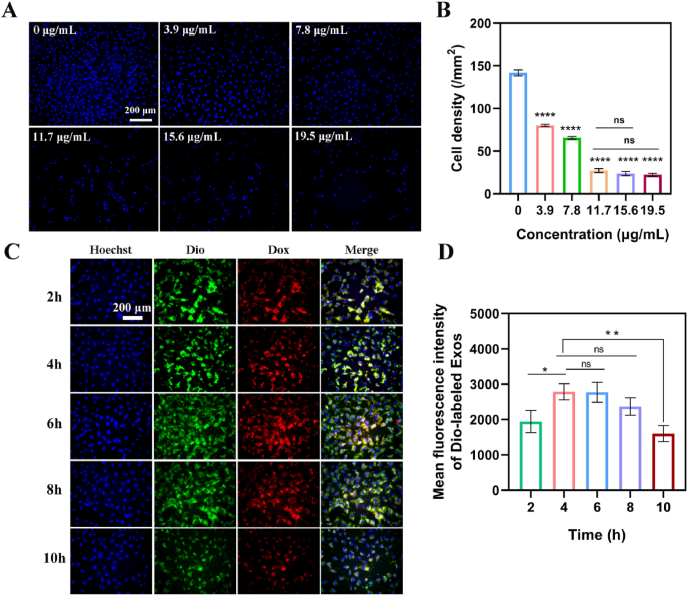
In vitro apoptosis and cell co-incubation assay. A-B) Representative fluorescence microscopy images of HLECs treated with different concentration of Dox@Exos for 24 h and quantitative analysis of density of the residual HLECs; C-D) Representative fluorescence microscopy images of HLECs co-incubated with Dox@Exos for different periods of time and quantification of fluorescence intensity. Hoechst 33,342-labeled nucleus: blue, Dio-labeled Exos: green, Dox fluorescence: red. *p < 0.05; **p < 0.01; ****p < 0.0001; ns, not significant.

To determine the optimal concentration of loaded Dox for good anti-proliferation effect, free Dox and Dox@Exos with different concentrations of Dox were co-incubated with the HLECs for 24 h. The cell density of each group was different after treated with varied Dox concentration and exhibited concentration-dependent anti-cell proliferation effect (Fig. 3A). Combined with the results of quantitative cell density analysis (Fig. 3B), we can find that there was no significant improvement of inhibition effect when the concentration of Dox was higher than 11.7 μg/mL. Therefore, the optimal concentration of Dox loaded in exosomes was determined to be 11.7 μg/mL, which was used in latter experiments. In addition, comparing with free Dox, Dox@Exos showed a better anti-proliferation effect (Fig. S3). The higher anti-proliferative effect of Dox@Exos is mainly due to the phospholipid bilayer structure of exosomes and their special membrane proteins like CD9, CD9 can directly promote the membrane fusion between exosomes and targeted cells and enhance cellular delivery of therapeutic drugs [41].

To optimize the co-incubation time between Dox@Exos and HLECs, cellular uptake of Dox@Exos by HLECs was also observed by fluorescence microscopy. As shown in Fig. 3C, facile uptake of Dox@Exos by HLECs was observed, and the fluorescent intensity of Dox@Exos in HLECs obviously increased with the incubation time. After 4 h of incubation, quantitative data in Fig. 3D revealed that about 1.43-fold increase of the Dio fluorescence intensity in HLECs was achieved compared with that of 2 h incubation, while no significant difference with that of 6 h incubation was observed. Interestingly, the mean fluorescence intensity began to decrease after 8 h of incubation. These is probably because the maximum uptake capacity was achieved after 4 h of incubation and the Dox@Exos taken by the cells began to release Dox and kill the cells, resulting in a decrease of cellular viability and cell density.

### 3.3 Enhanced cellular uptake and homologous targeting analysis in vitro

**Fig. 4:**
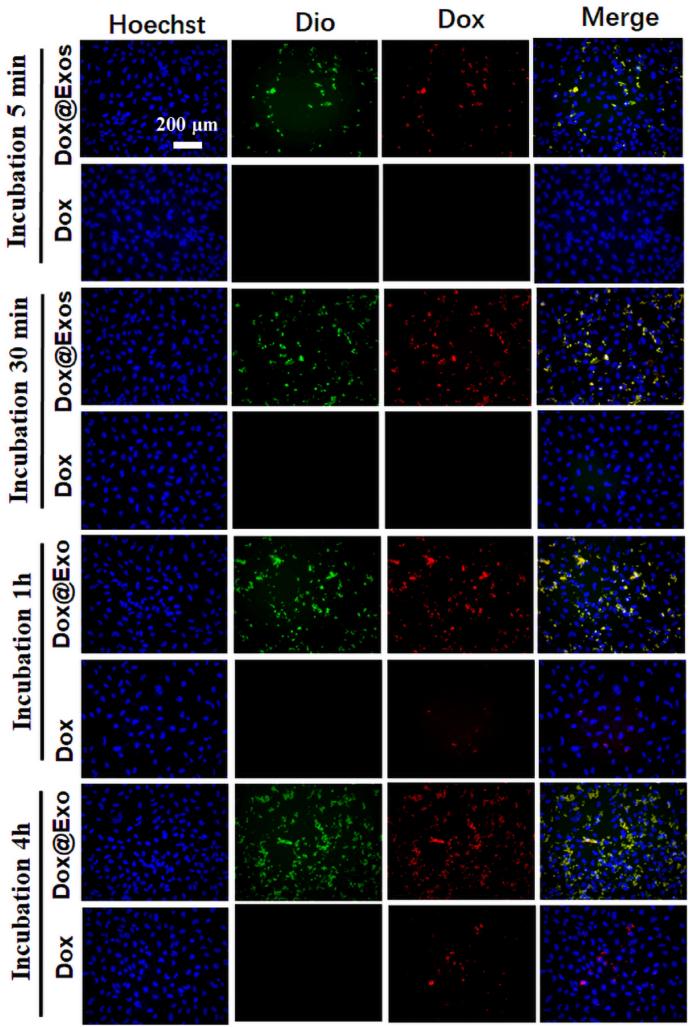
Enhanced cellular uptake. Representative fluorescence microscopy images of HLECs co-cultured with Dox@Exos and free Dox (equal to Dox 2.25 μg/mL) after 5 min, 30 min, 1 h and 4 h, respectively.

It is well-acknowledged that cellular uptake capacity is critical to the efficiency of drug delivery. According to previously published reports, exosomes with different sources exhibit diverse uptake outcomes as for different cells [36,50]. The HLECs were co-incubated with free Dox and Dox@Exos containing the same concentration of Dox. The intracellular trafficking of both Dox (red) and Dio-labeled Exos (green) was tracked by fluorescence microscopy at different time points. As shown in Fig. 4, after incubation with HLECs for 5 min, efficient cellular uptake of Dox@Exos was observed. The yellow dots (overlap of red and green fluorescence) indicated that the co-localization ratio of Dox and Exos was relatively high, suggesting that Dox was still encapsulated in Exos. However, either red fluorescence or green fluorescence was not observed when HLECs were treated with free Dox. As the incubation time increased, Dox and Dio-labeled Exos were increasingly concentrated in the cytoplasm, indicating that cellular uptake of Dox@Exos via cell endocytosis were enhanced. After 1 h of co-incubation, free Dox gradually entered the cytoplasm, and the red fluorescence of Dox became brighter with the incubation time. This is because small molecules can pass through membrane through a passive diffusion mechanism. Therefore, homologous exosomes had better affinity with their parent cells and performed higher delivery efficiency of drugs.

**Fig. 5:**
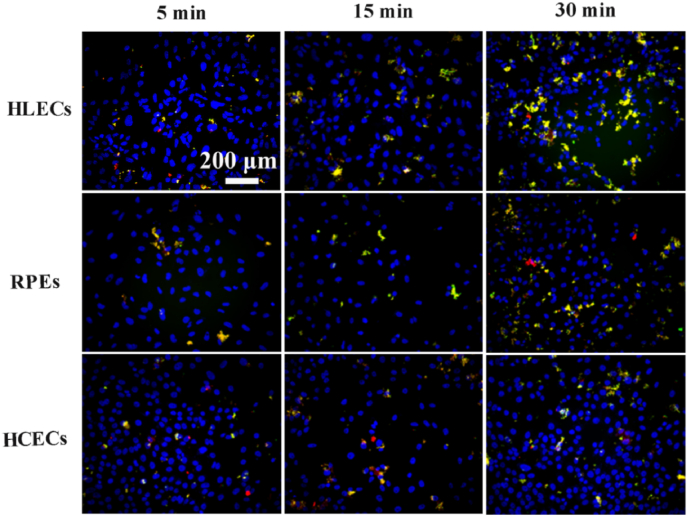
Homologous targeting analysis. Representative fluorescence microscopy images of HLECs, RPEs and HCECs co-incubated with Dox@Exos for 5 min, 15 min and 30 min, respectively. Blue color indicates cell nucleus, green color indicates Dio-labeled Exos, red color indicates Dox, yellow color is the overlap of green fluorescence and red fluorescence.

Consequently, the interactions between Dox@Exos (labeled with Dio) and diverse cells, including HLECs, RPEs and HCECs, were observed by a fluorescent microscope, so as to confirm the targeting of Exos to HLECs. As shown in Fig. 5, uptake efficiency of Dox@Exos by each cell type showed no significant difference during the initial 5 min, as indicated by the yellow fluorescence (overlap of red and green fluorescence). However, cellular uptake of Dox@Exos by HLECs was significantly enhanced with the incubation time, suggesting that exosomes displayed specific targeting behavior to HLECs.

### 3.4 Preparation and characterization of Dox@Exos-modified IOLs

**Fig. 6:**
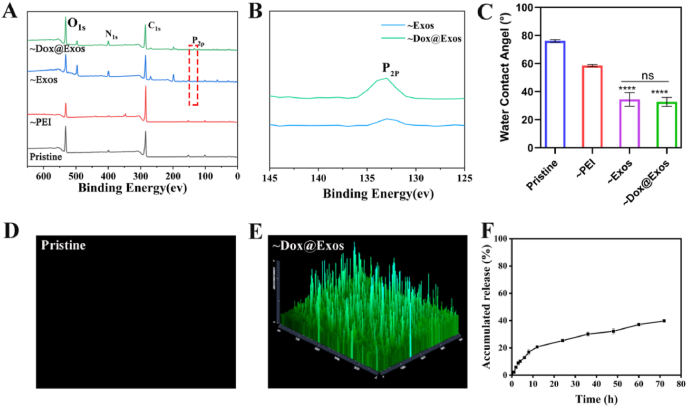
Preparation and characterization of Dox@Exos-modified IOL materials. A-B) Analysis of the surface elements by XPS during surface modification; C) Water contact angles of each sample; D-E) Fluorescence 3D images of pristine and Dox@Exos modified substrates; F) Cumulative release of Dox on the surface of Dox@Exos modified material. **p < 0.01; ****p < 0.0001; ns, not significant.

A polyethylene glycol terephthalate (PET) substrate was used to replace IOL materials when in vitro investigation. The negatively charged Dox@Exos was immobilized onto the positively charged aminated material surfaces via electrostatic assembling. The change of chemical elements during surface modification was analyzed by XPS. As shown in Fig. 6A and B, after Exos or Dox@Exos was deposited on the surface, owing to the special phospholipid bilayer structure of exosomes, characteristic peaks of phosphorus element appeared on the substrate surface, indicating that Dox@Exos was successfully immobilized. Meanwhile, water contact angle (WCA) measurements were also exploited to investigate the surface modification process. As shown in Fig. 6C, the WCA of pristine IOL material is about 76.1°, displaying a slightly hydrophilic state. Subsequently, the WCA of the aminated PET surface decreased to 58.5°. With the modification of Exos and Dox@Exos, the WCA of modified IOL surfaces decreases to 34.4° and 32.7°, which shows high hydrophilicity. The excellent hydrophilicity of the surfaces may provide a certain cell anti-adhesion effect [18,49,50]. At the same time, the change of WCA further confirms the successful prepared of Dox@Exos modified materials. The fluorescence 3D images (stained with Dio) in Fig. 6D and E demonstrated that, obviously, there was a large amount of uniformly dispersed green fluorescence on the surface of the Dox@Exos modified PET substrate while no fluorescence was observed on the surface of pristine PET, proving the successful deposition of Dox@Exos. Previous investigations have reported that the amount of exosomes immobilized onto the PEI precoating materials was controllable and exhibited the maximal loading capacity of materials, where dynamic equilibrium was obtained when the original exosome was 30 μg [47]. Herein, the exosomes solution used for adsorption contains 34.34 μg of exosome (Fig. S5), a large number of exosomes could be absorbed onto the surface of IOL. As shown in Fig. S5, the concentration of the exosome decreased from 0.1718 mg/mL to 0.1252 mg/mL after surface modification, which was detected by Enhanced BCA Protein Assay (BCA) Kit. Thus, the loading efficiency of Dox@Exos on the surface of the modified material was calculated to be approximately 27.1%. Based on the above situation, the final density of exosomes on the IOL surface reached 32.9 μg/cm^2^. In addition, judging from the drug release behavior of the modified material, the coating showed a slow and continuous release (Fig. 6F). With regard to the stability of the incorporated exosomes on the material surface, it is interesting that two opposite phenomena have been reported in the previous publications. Some investigations demonstrated that the exosomes are stable on the surface whereas some investigations revealed that exosomes can be released from the coating in some extent [47,51,52]. In view of this phenomenon, we also monitored the stability of exosomes on the Dox@Exos coating modified IOL surface in this study by testing the protein content in the releasing buffer. It was found that the release behavior of exosomes was similar to that of Dox, indicating that the released Dox were all caused by the shedding of Dox@Exos rather than the rupture of exosomes (Fig. S6A). This also provides a practical basis for targeted treatment of PCO. At the same time, particle size detection showed a particle size distribution of 356 nm in the released supernatant, which may be due to partial aggregation of the shed exosomes, and the exosome structure could also be observed by transmission electron microscopy (Figs. S6B–C). All these above proved that the Dox@Exos modified IOL prepared in this study showed slow drug release behavior.

### 3.5 Surface morphology and optical properties

**Fig. 7:**
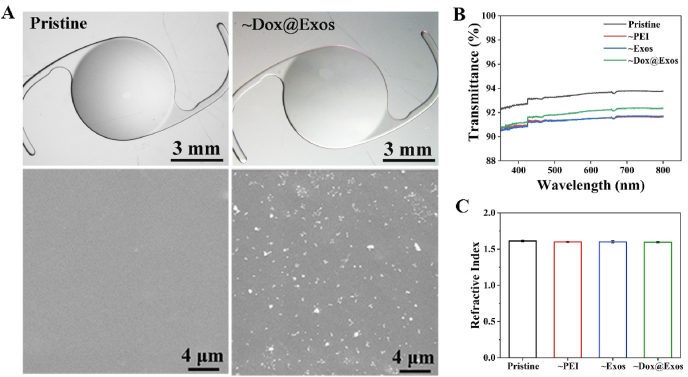
Surface morphology and optical properties. A) Macrotopography and microtopography of the IOL surface before modification (pristine IOL) and after modification (Dox@Exos-IOL); B–C) Transmittance and refractive index of the modified IOLs.

As artificial refractive tissues implanted in eyeballs, the surface morphology and optical properties of IOL are of great importance. Thus, SEM and type microscope were used to observe the morphology of the modified IOLs. As can be seen from Fig. 7A, the pristine IOL is colorless and transparent, while the surface of Dox@Exos modified IOL is a light red with good transparency. SEM images revealed that Dox@Exos with a diameter of about 100 nm were uniformly distributed on the surface of IOL, indicating that the Dox@Exos were successfully immobilized through electrostatic interaction. As shown in Fig. 7B and C, although the IOL transmittance decreased slightly after surface modification, the light transmittance was still above 90% within the visible spectrum. Furthermore, the refractive index remained almost unchanged after surface modification. All these results demonstrated that surface modification with Dox@Exos caused no significant change on surface morphology and optical properties of IOL.

### 3.6 In vitro antiproliferative analysis

**Fig. 8:**
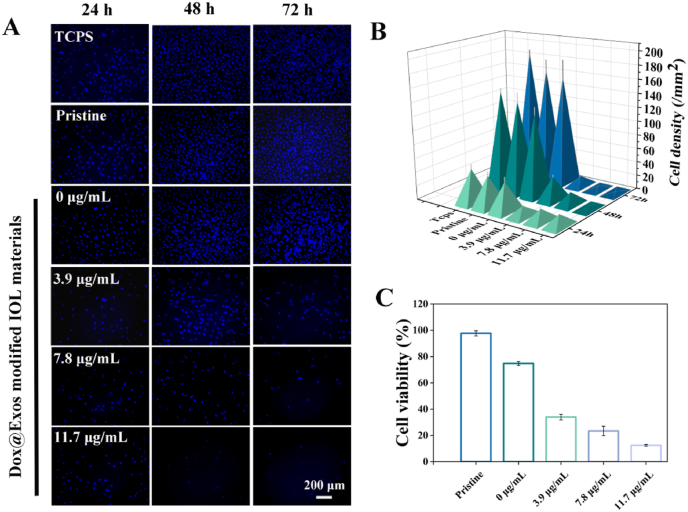
In vitro antiproliferative analysis. A) Fluorescence microscopy images of HLECs co-incubated with Dox@Exos modified substrate for 24 h, 48 h and 72 h, respectively; B) Corresponding quantification of the residual HLECs density; C) CCK-8 assays after various treatments.

The residual HLECs in the capsular bag after cataract surgery proliferated, migrated and differentiated on the surface of the lens, eventually leading to PCO. However, after the implantation of a Dox@Exos modified IOL, the proliferation of the residual HLECs in the postoperative capsule can be effectively inhibited by Dox released from exosomes. The pristine substrate, exosome modified substrate and Dox@Exos modified substrate were placed in 96-well plates and co-cultured with HLECs for 24, 48 and 72 h, respectively. Cellular apoptosis was observed by fluorescence microscopy after Hoechst 33,342 staining. Cell growth status was mainly evaluated by the density of nucleus on the material surface. The fluorescence micrographs in Fig. 8A and quantitative analysis results in Fig. 8B showed that with the prolongation of time, the cell density increased and there was no significant difference among the polystyrene tissue culture plate (TCPS) group, the substrate group and the exosome modified group, demonstrating good cytocompatibility of exosomes. On the contrary, the cell density of the Dox@Exos modified substrate group was significantly reduced. CCK-8 results in Fig. 8C further confirmed the anti-proliferation effect of Dox@Exos modified IOL. All these results comprehensively proved that the Dox@Exos modified IOL significantly inhibited the proliferation of HLECs.

### 3.7 In vivo anti-PCO efficacy and PCO analysis

**Fig. 9:**
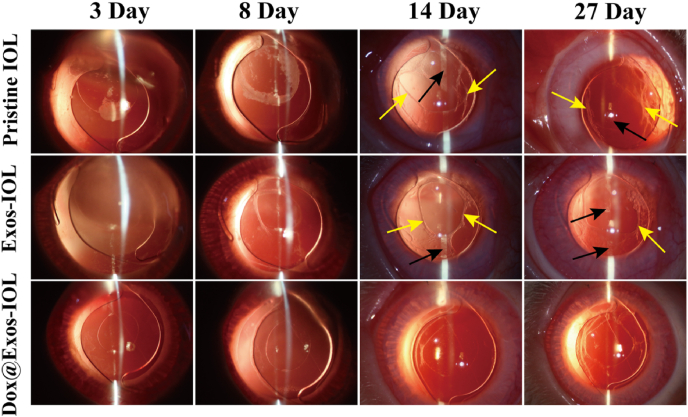
Slit lamp microscopy observation of PCO in pristine IOL group, exosome-modified IOL group and Dox@Exos-IOL group at 3 days, 8 days, 14 days and 27 days; black arrows indicated the proliferating cells, and yellow arrows were introduced to refer to capsular wrinkles.

After the implantation of the Dox@Exos modified IOL, a slit lamp was used to observe the occurrence and development of PCO at specific time points (3, 8, 14, 27 days). As shown in Fig. 9, in the first week after surgery, the pristine IOL implantation group exhibited the most serious anterior chamber exudation while just slight exudation was observed for the Dox@Exos modified IOL group. For all groups, corneal edema occurred in the early postoperative period and disappeared after one week. Moreover, the Dox@Exos modified IOLs were the cleanest and the most transparent, demonstrating that the Dox@Exos modified IOL had good biocompatibility. As expected, the posterior capsule hyperplasia starts to occur in control and Exos-IOLs groups as early as 2 weeks postoperatively. In the second week, judging from the proliferation of LECs on IOLs surfaces and the position irradiated by the slit lamp, the mouth of capsules began to shrink and thicken and a large number of folds appeared in the capsules and gradually approached the central optical zone. The blank arrows indicated the proliferating cells on the IOL surface were tiled and granular, which appears gray white under the irradiation of the slit lamp. The yellow arrows indicated the capsular wrinkles which may be caused by the massive deposition of extracellular matrix produced by cell proliferation. During the investigation periods, the PCO in control and Exos-IOLs groups became more serious as time increasing. In contrast, the posterior capsule for the Dox@Exos modified group was not significantly turbid, and the optical zone was clear and transparent, indicating a significantly enhanced PCO inhibitory effect.

**Fig. 10:**
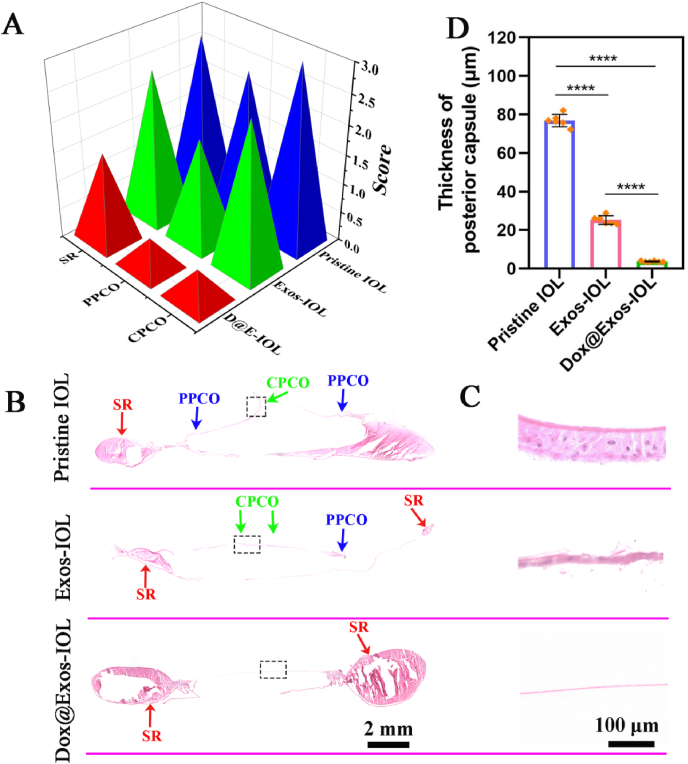
In vivo anti-PCO efficacy. A) PCO scores of various IOL groups (CPCO, the 3 mm diameter pouch in the optical center; PPCO, the 6 mm diameter pouch in the optical region except the central part; SR, the peripheral part of the capsule); B) inhibitive efficiency evaluation of various IOLs via HE staining of capsule after surgery on days 27; C) Enlarged images of blank box selection of posterior capsules in each group 27 days after surgery; D) Corresponding quantification of posterior capsules thickness. ****p < 0.0001.

After the observation processes, the rabbits were euthanized and the lens capsule, cornea, iris and retina were carefully isolated. To further understand the severity of PCO, the capsules were stained with HE and observed. As shown in Fig. 10B, the capsular tissue section in pristine IOL and Exos-IOL group showed that a lot of hyperplasia appeared in the capsule center and formed thick multicellular fibrous membrane, which are identified as CPCO and PPCO as indicated by green arrows and blue arrows respectively. However, almost no hyperplasia was found in Dox@Exos-IOLs group. Combined with the enlarged picture of the posterior capsule in each group, the posterior capsule of control and Exos-IOL groups were significantly thickened, indicating severe cell proliferation and serious PCO development, while the Dox@Exos group did not (Fig. 10C), revealing that Dox@Exos-IOLs effectively inhibited the development of PCO. After statistical analysis of the average thickness of each part of the posterior capsule in each group, it further confirmed that the proliferation of the posterior capsule was significantly inhibited after Dox@Exos-IOL implantation (Fig. 10D).

As mentioned above, the severity of PCO can be graded according to the development of PCO: central posterior capsular opacification (CPCO, the 3 mm diameter pouch in the optical center), peripheral posterior capsular opacification (PPCO, the 6 mm diameter pouch in the optical region except the central part), Soemmering's ring (SR, the peripheral part of the capsule) and no PCO occurs [8,[13], [14], [15]]. The severity of PCO in each level was assessed on a scale of 0–3 points. The higher the score is, the more severe PCO developed. As shown in Fig. 10A, the pristine IOL group exhibited severe PCO with the scores of CPCO, PPCO and SR of 3, 2.50 ± 0.57 and 2.75 ± 0.5 respectively. Similarly, the Exos-IOL group also scores highly of 2.50 ± 0.57, 1.75 ± 0.5 and 2.5 ± 0.57, respectively. On the contrary, there is no significant PCO occurrence in Dox@Exos-IOL group except some SR appearing with the score of 1.5 ± 0.57. Collectively, all these results proved that the Dox@Exos modified IOL significantly inhibited the occurrence and development of PCO through releasing anti-proliferative drugs and killing the residual HLECs. Due to the proliferation of residual HLECs in the equatorial region after cataract surgery, SR occurred and was observed in all group as indicated by red arrows (Fig. 10B), which, in some respects, reflects the biosafety of such functionalized drug eluting coating modified IOLs, as it only kill cells that cling to the coating area, but is safe to the surrounding tissues [48]. Combined with the current research results, the modification of the IOL with drug-loaded exosomes eliminated only the cells between the optical part of the IOL and the anterior or posterior capsule. Therefore, the formation of SR proves its safety and targeting from another aspect.

### 3.8 In vivo biocompatibility evaluation

**Fig. 11:**
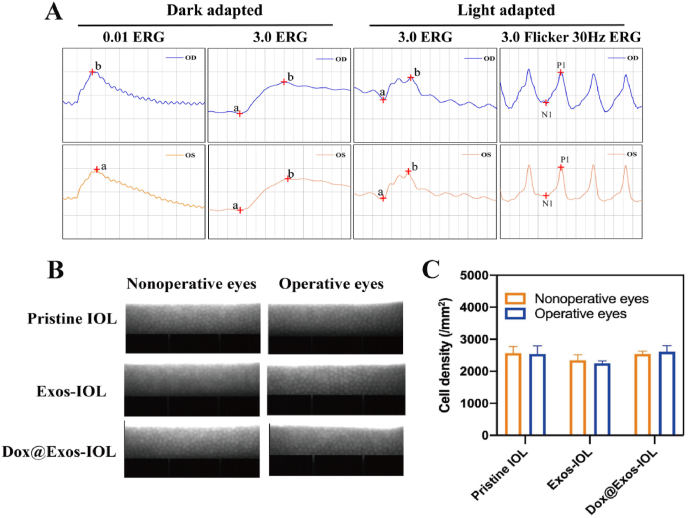
In vivo biocompatibility evaluation. A) ERG of normal (left eye, blue) and surgical eyes (right eye, orange) in Dox@Exos-IOL group. B–C) Representative specular microscopy images of corneal endothelial cells and the quantitative analysis of cornea endothelial cells.

**Fig. 12:**
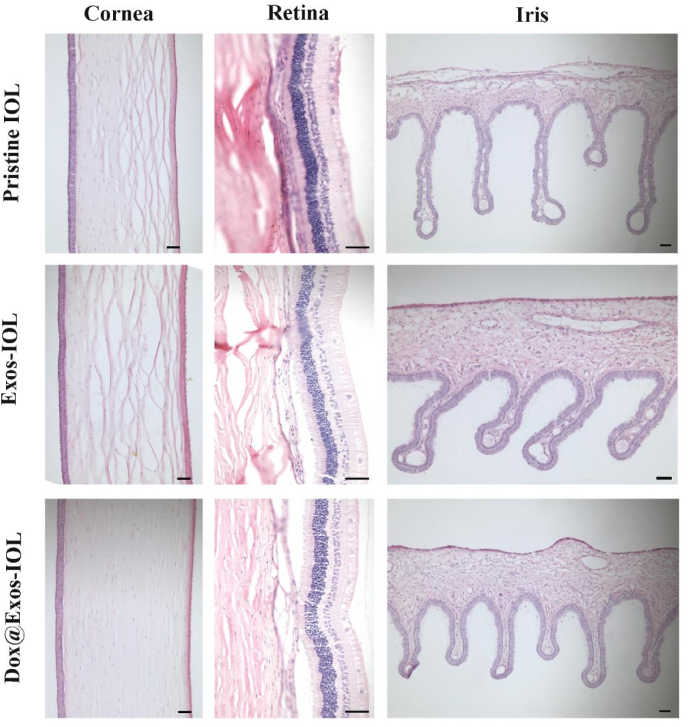
Representative images of HE stained tissue involving corner, retina and iris. The scale bar is 50 μm.

As an intraocular implant material, vivo biocompatibility and biosafety of the modified IOLs is of great importance. The influence of the IOLs on the retina function was evaluated by ERG. ERG is a means of detecting retinal function and optic nerve function through light adaptation or dark adaptation. As shown in Fig. 11A, by analyzing the ERG of normal eyes (left eye, blue) and surgical eyes (right eye, orange) in Dox@Exos-IOL group, it was found that the electrophysiology curves of left and right eyes were basically consistent in dark and light adaptation. The results showed that the implanted Dox@Exos-IOL had no effect on retinal function and optic nerve function. Furthermore, the specular microscopy images in Fig. 11B showed that the morphology and structure of corneal endothelium cells remain unchanged after the implantation of Dox@Exos-IOL. At the same time, there was no significant difference in corneal endothelial cell density among all groups (Fig. 11C), suggesting that Exos and Dox@Exos modified IOLs were safe to cornea. Besides, intraocular pressure, weight measurement and fundus photography were also performed in animal experiments. As shown in Figs. S8A–B, the implantation of Dox@Exos-IOL does not affect intraocular pressure (IOP) compared with the preoperative IOP, and there was no significant fluctuation in body weight, indicating that the rabbits grew well during the experiment. The fundus photographs in Fig. S8C indicate that the optic disc and the blood vessel are visible in each group and no obvious abnormality was observed, further confirming Dox@Exos-IOL is highly biosafe to the eye. Pathological tissue section is an important method to evaluate the safety of IOL. The typical staining of cornea, iris and retina slices of each group presented in Fig. 12. Demonstrated that all tissues were in normal shape and complete in structure. All above results demonstrated that Dox@Exos-IOL exhibited good intraocular biocompatibility, providing a promising potential for the further application.

## 4 Discussion

In summary, an exosome-functionalized drug-loaded IOL with excellent biocompatibility was developed for efficient PCO prevention based on the homologous targeting of exosome. An antiproliferative drug, Dox, was encapsulated into exosome through electroporation and Dox@Exos was subsequently immobilized on the surface of IOLs through electrostatic interaction. UV–Vis, XPS, and SEM measurements demonstrated that Dox@Exos modified IOL was successfully constructed, which still maintained excellent optical properties. The in vitro analysis revealed that Dox@Exos showed more efficient cellular uptake and targeted delivery, resulting in efficient apoptosis of the residual LECs. In vivo animal investigations indicated that the Dox@Exos-IOLs exhibited superior inhibitory effect on the development of PCO and excellent intraocular biocompatibility. We believe this work will provide insightful guidance in surface modification of IOLs with a targeted drug delivery coating for effective PCO prevention.

## 5 Declarations of competing interest

The authors declare that they have no financial and personal relationships with other people or organizations that could have appeared to influence the work reported in this manuscript.

## CRediT authorship contribution statement

**Siqing Zhu:** Investigation, Data curation, Formal analysis, Investigation, Writing – original draft, preparation. **Huiying Huang:** Investigation, Methodology. **Dong Liu:** Investigation. **Shimin Wen:** Investigation. **Liangliang Shen:** Writing – original draft. **Quankui Lin:** Conceptualization, Project administration, Resources, Supervision, Writing – review & editing.

## Declaration of competing interest

The authors declare that they have no known competing financial interests or personal relationships that could have appeared to influence the work reported in this manuscript.
