# Supplementary material for: Augmented cellular uptake and homologous targeting of exosome-based drug loaded IOL for posterior capsular opacification prevention and biosafety improvement
Source: Bioact Mater. 2022 Feb 26;15:469–81. doi: 10.1016/j.bioactmat.2022.02.019 (PMC8958386; doi:10.1016/j.bioactmat.2022.02.019)
Supplement: Multimedia component 1 [file mmc1.docx]

Augmented cellular uptake and homologous targeting of exosome-based drug loaded IOL for posterior capsular opacification prevention and biosafety improvement

Siqing Zhu, Huiying Huang, Dong Liu, Shimin Wen, Liangliang Shen, Quankui Lin*

Department of Biomaterials, School of Ophthalmology & Optometry, Eye Hospital, Wenzhou Medical University, Wenzhou 325027, P. R. China

*Corresponding Author: E-mail: linqk@wmu.edu.cn


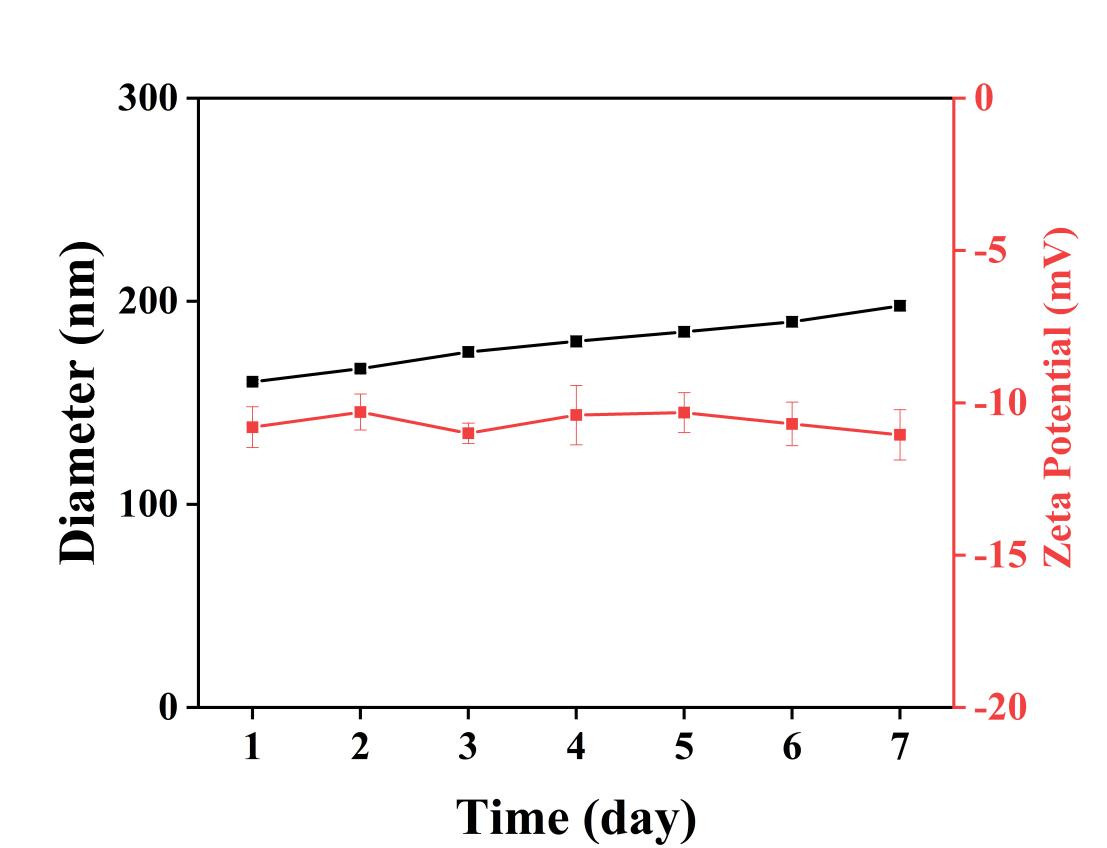


Figure S1. Stability of Dox@Exos. The diameter and Zeta Potential of Dox@Exos in PBS within 7 days.


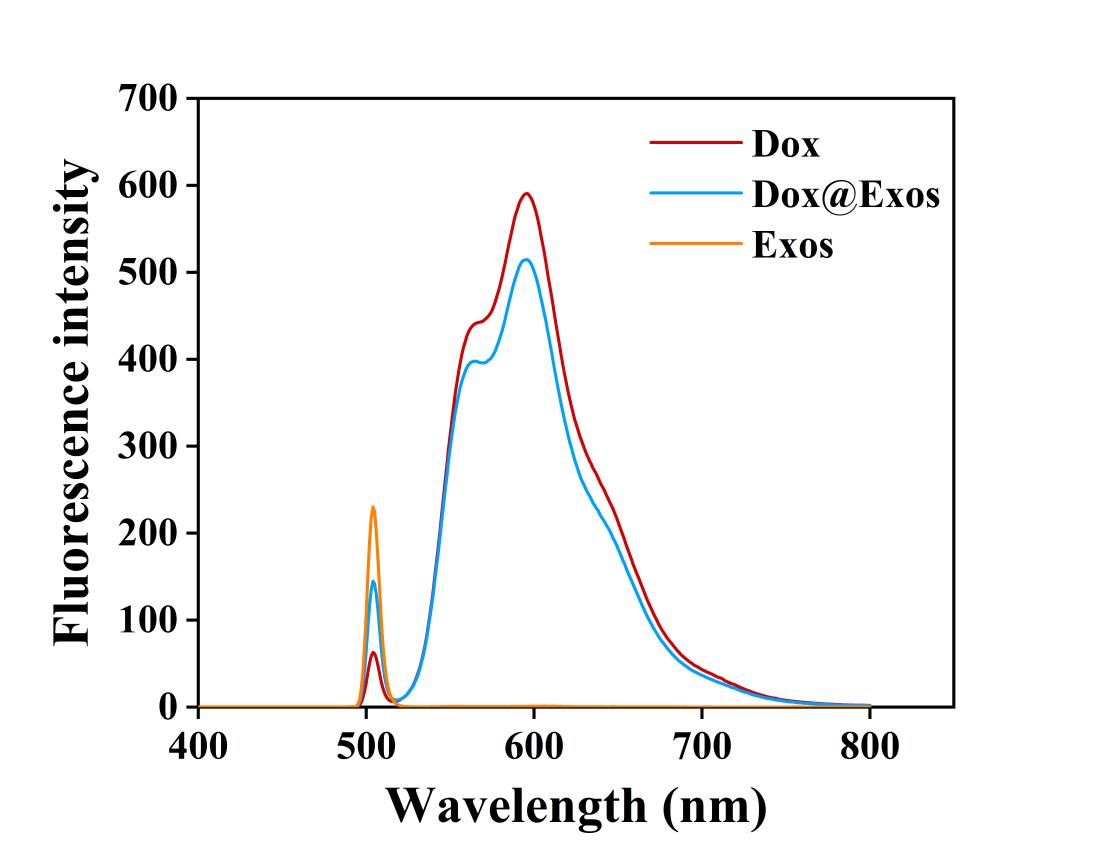


Figure S2. [Fluorescence spectrum](C:/Users/19759/AppData/Local/youdao/dict/Application/8.10.3.0/resultui/html/index.html#/javascript:;) of exosomes, free Dox, and Dox@Exos dispersed in PBS.


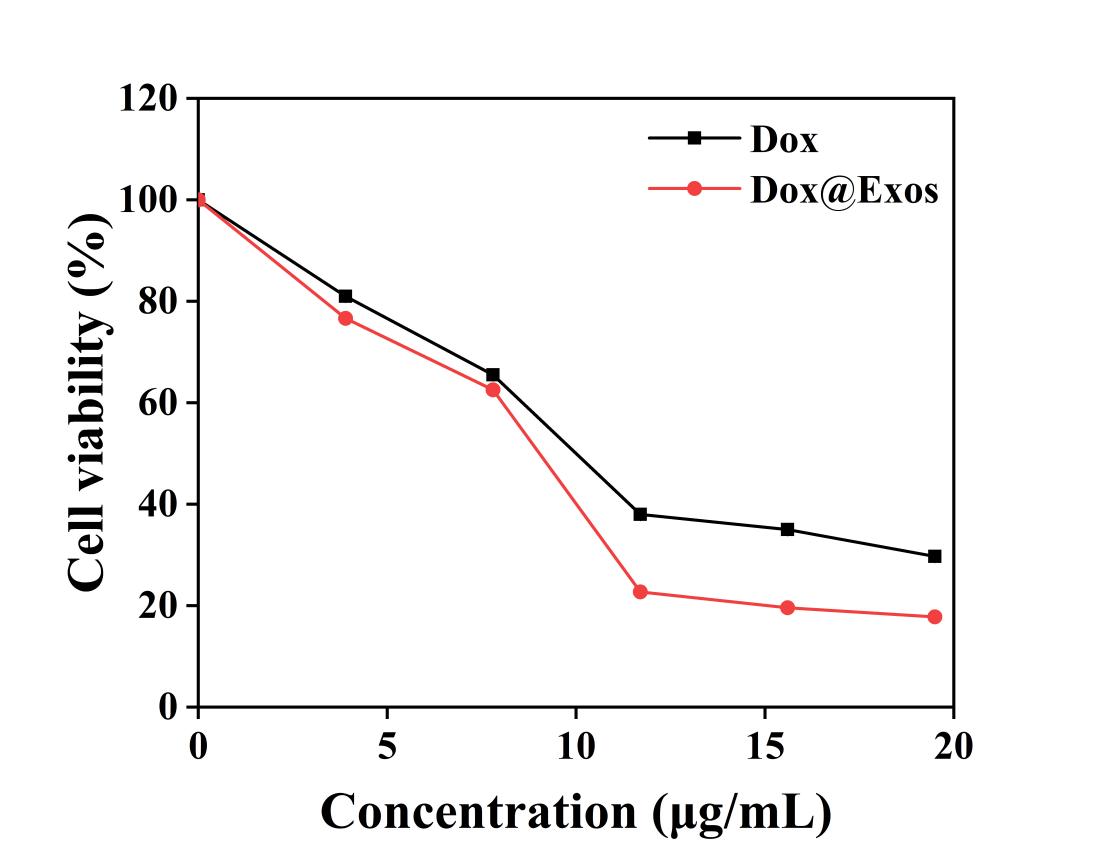


Figure S3. Relative viabilities of HLECs after being incubated with Dox@Exos and Free Dox for 24 h.


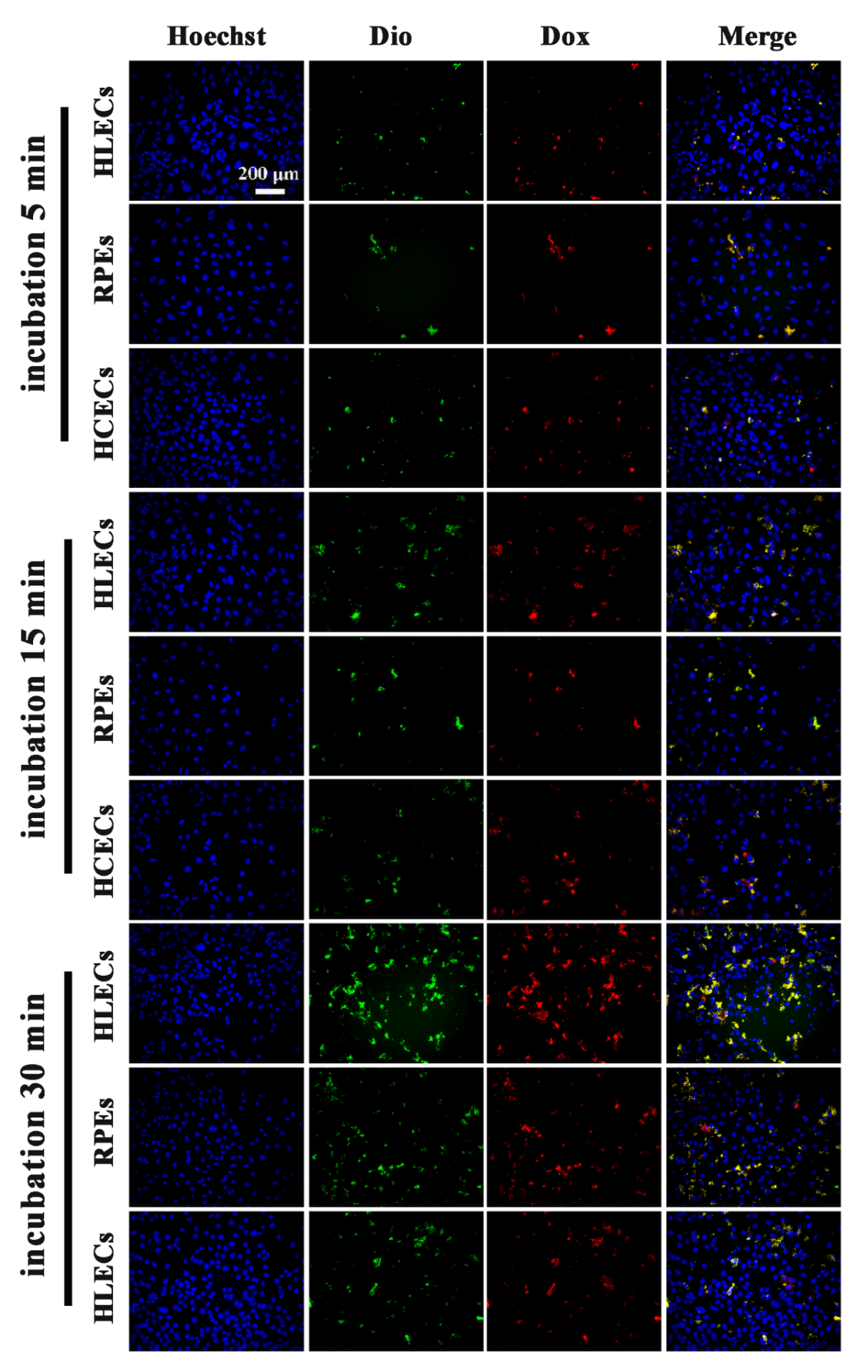


Figure S4. Homologous targeting analysis. Representative fluorescence microscopy images of HLECs, RPEs and HCECs co-incubated with Dox@Exos for 5 min, 15 min and 30 min, respectively. Blue is cell nucleus, green is Dio-labeled Exos, red is Dox, yellow is the overlap of green fluorescence and red fluorescence.


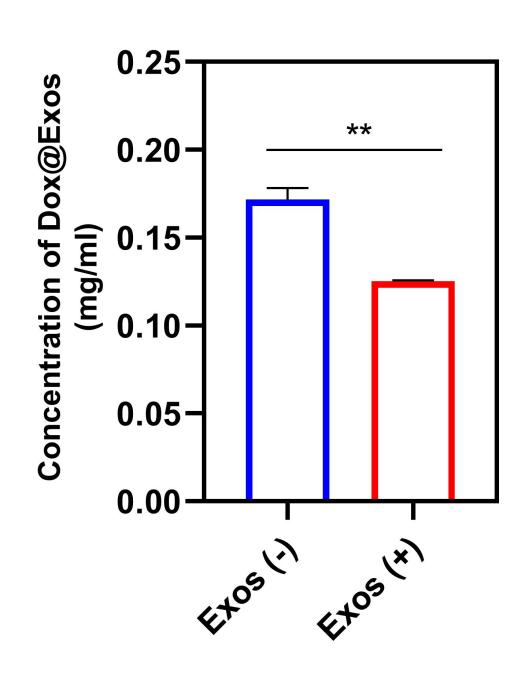


Figure S5. The difference of concentration of Dox@Exos in suspension before and after the Dox@Exos immobilized on materials.


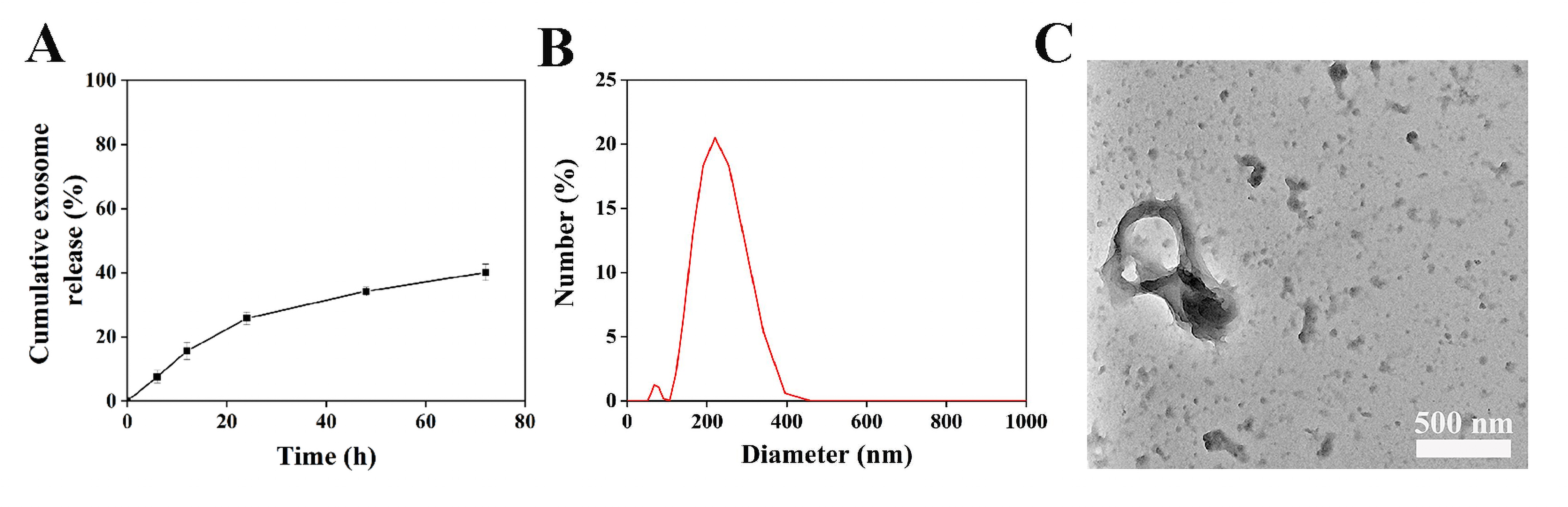


Figure S6. Study on exosomes release behavior of Dox@Exos modified IOL. A) cumulative exosome release amount of Dox@Exos modified IOL; B) Particle size distribution of exosomes in release fluid; C) TEM images of exosomes in release fluid


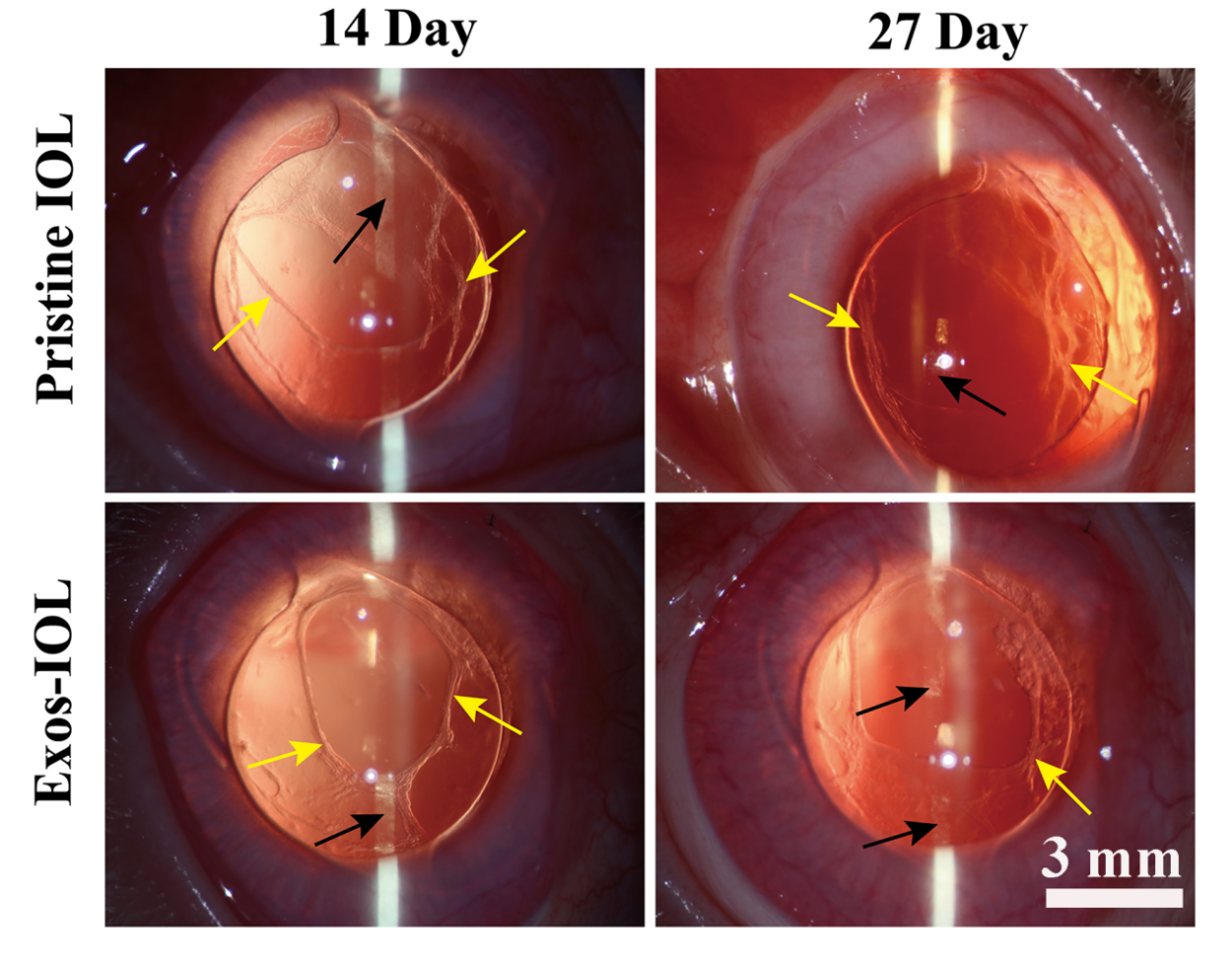


Figure S7. Slit lamp microscopy observation of PCO in pristine IOL group, Exos-IOL group in 14 days and 27 days; black arrows indicated the proliferating cells, and yellow arrows were introduced to refer to capsular wrinkles.


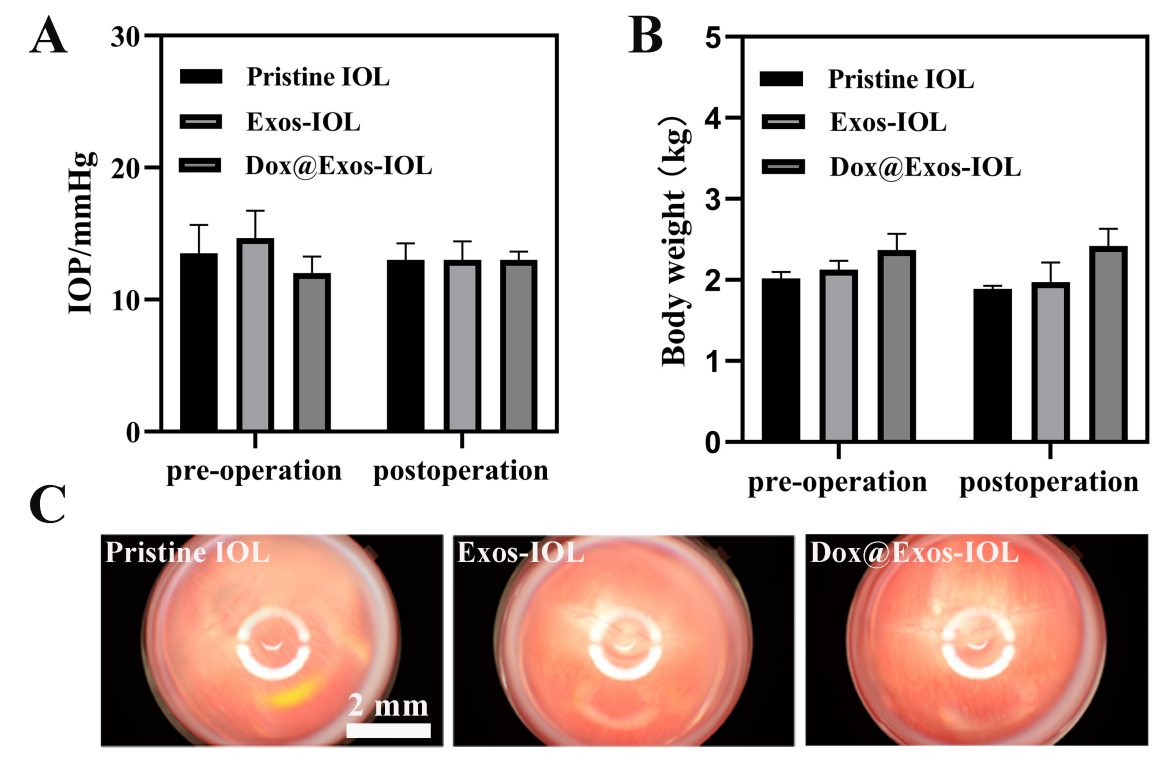


Figure S8. In vivo biocompatibility evaluation. A) Intraocular pressure changes in each group. B) Body weight of each group before and after operation. C) [Eye-ground](C:/Users/19759/AppData/Local/youdao/dict/Application/8.10.3.0/resultui/html/index.html#/javascript:;) [photography](C:/Users/19759/AppData/Local/youdao/dict/Application/8.10.3.0/resultui/html/index.html#/javascript:;) of each group after surgery on days 27.
